# The Influence of Personal Evaluation and Social Support on Career Expectations of College Students

**DOI:** 10.3390/bs13120992

**Published:** 2023-11-30

**Authors:** Rui Wang, Mengru Wang, Georgi V. Georgiev

**Affiliations:** 1 School of Educational Science and Technology, Anshan Normal University, Anshan 114007, China; wangr1981@163.com; 2 Center for Ubiquitous Computing, University of Oulu, 90570 Oulu, Finland; mengru.wang@oulu.fi

**Keywords:** career expectations, college students, personal evaluation, social support, self-development, welfare, gender, major

## Abstract

This study aimed to investigate the factors influencing career expectations, determine the influence of college students’ personal ability on personal evaluation in the process of gaining employment, and further explore the impact of personal evaluation and social support on career expectations. This study used a random sampling method to administer questionnaires to final-year undergraduates majoring in the liberal arts, science, art, and sports at two Chinese universities. Career expectations were positively correlated with satisfaction. The preferred employer for graduates is a school. In selecting a career, college students believed that exerting their talents was most important. Personal evaluations had a significant effect on self-development in career expectations. The level of social support had a significant effect on prestige and welfare stability in career expectations.

## 1. Introduction

Occupation is a fundamental factor in the quality of life, experience of being human, and social change in individuals and the societies to which they belong [1]. Career expectations refer to an individual’s desire to obtain material and spiritual satisfaction through a certain occupation [2]. Career expectations directly affect people’s career choices and affect their entire lives. Specifically, career expectations mainly include health factors (such as wage income, welfare benefits, working environment, and working conditions), prestige factors (such as the geographical location and popularity of the employer, the social status of the work, and social value and reputation in society), and development factors (such as being able to work independently, equal opportunities in work, fair competition and personal abilities, and strengths) [2]. Betts has published a report on the income expectations of college students [3], and Dominitz and Manski have conducted a cooperative study on the income expectations of high-school and college students [4]. Their studies reached the same conclusion: college students generally overestimate their expected income; there are considerable differences in their expected levels of income, and income expectations are affected by gender, family background, school, grade, major, achievement, source of knowledge, and other factors. Undergraduate students generally do not form mature ideas about income expectations until their fourth year [3,4].

## 2. Background

People often associate the term “personal evaluation” with psychologically oriented supervision, in which individuals or groups are encouraged to self-evaluate to clarify their goals, motivations, and likelihood of improving performance [5]. Personal evaluation provides self-enhancement, and the self protects itself from negative information by selectively processing positive information [6]. Self-evaluation is based on the results of interactions between a subject and others. These include social comparison, the evaluation of others, and self-assessment [7]. Students with positive self-competence assessments have significantly better achievement motivation, learning engagement, and academic performance than those with negative assessments of self-competence [8]. Informal social support and self-esteem significantly enhance positive mental health among college students [9].

Social support is defined as information that leads individuals to believe that they are cared for, loved, and respected and are members of a network of mutual obligations. It involves supportive interactions between people and protects against the health effects of life stress [10]. Factors affecting social support typically depend on gender and family. Generally, women are more supportive, nurturing, and emotionally connected compared to men. Thus, they are more capable of providing support but are more dependent on social support for their mental health [11]. Matud et al. [12] found that women perceived global social support while men perceived emotional and instrumental support as distinct factors. Regarding choosing parental and peer support, women are more likely to choose peer support than men and have higher levels of stress and physical and mental health problems [13]. Ding et al. found that the level of social support for female students was significantly higher than that for male students, which was mainly reflected in two aspects: subjective support and utilization [14]. Cheng found that female students demonstrated significantly higher levels of social support, satisfaction, information support, companionship support, material support, and emotional support than male students [15]. This study posits two reasons for the different results in the studies of social support among men and women. The first is that the difference in the measurement tools has not been studied, and the second relates to the limitations of the research method or sampling. Additionally, on a micro social level, support from family and friends significantly predicts psychosocial maturity levels [16]. The lower the parental support is, the stronger the entity thinking and the higher the anxiety are [17].

Previously, research has highlighted how social conditions shape career expectations [18,19]. Developing students’ self-efficacy to handle risks along with college satisfaction can empower more hopeful visions about jobs post-graduation [18]. It has been suggested that personal evaluations and social support have an impact on career expectations. Metheny and McWhirter [19] have found that family status and support are associated with career decision self-efficacy and outcome expectations. Xia et al. [20] have found that career social support positively affects career adaptation and employability, with career adaptation mediating the relationship between social support and employability [20]. The provision of career counseling services has been found to favorably impact an individual’s vocational self-concept [21]. Vocational self-concept has a notable positive impact on employment competencies.

The research highlights the significance of career expectations through career barriers and self-efficacy in career decision-making as influences on students’ college adjustment, even amidst the pandemic [22]. It points to the need to support students’ career development and decisions to improve adaptation [22]. The career expectations, including advancement prospects, income potential, and business opportunities, are important factors driving students’ choice of major, especially for those selecting STEM fields [23]. Family communication and support can promote women’s participation in STEM fields, like engineering, even when broader cultural discourses may discourage it; families can play important roles in shaping women’s STEM career choices [24]. Students find information about career prospects helpful for deciding on a college major. Career advancement was an important factor influencing students’ choice of college major, with over two-thirds of students rating it as highly or moderately influential [23]. Students seem to consider the potential for career growth when selecting their major. For example, the persistence of students’ aspirations for the American Dream, contrasted with their disillusionment regarding the inability of their college degrees to secure attractive and fulfilling employment opportunities, is a notable pattern [25].

Furthermore, previous studies have highlighted the importance of social support through career counseling to help students overcome difficulties, build self-concept, and match their skills/interests to career expectations, given the social climate in South Korea [26]. The career expectations are shaped by social factors like the geographic background [27]. In this context, improving self-concept and finding purpose in a career can help students overcome difficulties and have realistic career expectations.

Previous studies have suggested that social support positively impacts college students’ career expectations in China. Social support is also associated with career adaptability among Hong Kong university students [28]. Wang and Fu [29] have further supported these findings by showing that social support enhances career adaptability among Chinese college graduates [29]. Furthermore, social support from both family and peers is negatively correlated with stress and positively correlated with academic competence, social acceptance, and desire to continue education [30].

Such findings have indicated a positive and direct relationship between self-concept and occupational choice intention [31]. Social support is positively and directly associated with academic performance. However, the effects of self-concept and social support on occupational choice intention, when mediated by academic performance, are not statistically significant [31]. Franco et al. [32] have revealed a significant negative association between stress levels, social support, and career outcome expectations [32]. Furthermore, reduced social support plays an indirect and partial role in elucidating the association between stress and outcome expectations. Such findings indicate that to enhance the career outlook of international students, it is imperative for counselors to not only address stress but also recognize its impact on the availability of social support within the host country [32].

However, employment pressure caused by the expansion of college enrollment, and thus an increase in the number of college graduates, has intensified, and graduates face different challenges. The problem of college students’ career choices has always been the focus of scholarly research, which focuses on professional values, professional ability, and professional interest. However, the “expectation problem” in students’ career choices is a new research topic. Therefore, this study had four main objectives. First, we investigated the factors influencing career expectations. Second, we determined the influence of college students’ personal abilities on personal evaluations in the process of gaining employment. Third, we explored the impact of personal evaluations and social support on career expectations. Fourth, we discussed the influence of social support on career expectations.

Our study had three hypotheses:

**H1.**  *Students of different genders and majors have different career expectations*.

**H2.**  *Students’ career expectations are related to their genders and majors.*

**H3.**  *There is a correlation between personal evaluations, social support, and students’ career expectations.*

## 3. Methods

### 3.1. Participants

This study used random sampling to recruit 380 final-year undergraduates at the Northeast Normal University and Anshan Normal University in China and distributed questionnaires. All participants provided informed consent to participate in the study. Participants majored in liberal arts, science, art, or sports. A total of 365 questionnaires were returned, of which 322 were deemed valid on the basis of completeness. Of the total participants, 151 were from the Northeast Normal University, 171 were from the Anshan Normal University, males accounted for 33.11%, females accounted for 66.9%, liberal arts majors accounted for 54.3%, science majors accounted for 35.8%, and art and sports majors accounted for 9.9%.

### 3.2. Tools

Four questionnaires were used for comprehensive investigation and analysis. SPSS statistical software (SPSS Statistics 29) was used for correlation analysis, principal component analysis, t-tests, parametric statistics, and other statistical analyses.

#### 3.2.1. Basic Questionnaire

A basic questionnaire was developed based on the preliminary survey, which included questions such as “can you describe your outlook”, “are you satisfied with your major”, and “what kind of organizations do you intend to join?” The questionnaire includes 10 questions concerning demographic variables, professional satisfaction, career prospects, graduation plans, and trends in choosing employers.

#### 3.2.2. Career Expectations Scale

On the basis of research on professional values, this study employed the career expectation scale utilized in a previous study by Wu [33]. This questionnaire asks participants to rank each career expectation item according to importance, reflecting the degree of importance that students attach to the various factors. The responses are scored on a five-point Likert scale ranging from “1 = not important” to “5 = very important”. The 22 variables on the Career Expectations Scale are divided into three factors: prestige, self-development, and stability. Among them, the prestige factors include eight variables related to status, such as an employer’s high visibility, ease of gaining distinction and starting a family, and opportunities for promotion. The self-development factors include eight variables related to personal development, such as the ability to utilize talent and challenging work. Stability factors include six variables related to the stability and welfare of the occupation.

#### 3.2.3. Personal Assessment Questionnaire

A personal questionnaire was prepared according to the Rating Scale for Mental Health to subjectively evaluate students’ self-confidence [34]. The questionnaire includes 54 items scored on four levels, with total scores ranging from 54 to 216. The higher the score is, the higher the degree of self-confidence is.

#### 3.2.4. The Social Support Scale

The Social Support Scale, designed by Xiao in 1986 and revised in 1990, was used [35]. This scale evaluates students’ actual support, subjective experience of support, and individual use of support. The mean score of the test scale obtained by participants was 34.56 with SD of 3.73, and the consistency of the two-month retest was 0.92, indicating that the questionnaire had good retest reliability. The Social Support Scale consisted of 10 items; items 2, 6, and 7 were objective support questions, and items 1, 3, 4, and 5 were subjective support questions. The remaining questions related to utilization of support.

## 4. Data Analysis Procedures and Results

### 4.1. Major Satisfaction

Of the 322 survey participants, males accounted for 33.11%, females accounted for 66.9%, students majoring in liberal arts accounted for 54.3%, science majors accounted for 35.8%, and art and sports majors accounted for 9.9%. These findings were in line with the enrollment ratio in most schools of this type.

The major satisfaction survey is a subjective evaluation of whether students are satisfied with their major. The project was divided into three majors: liberal arts, science, and art and sports. The project was divided into three grades: satisfaction, general satisfaction, and dissatisfaction, with scores of 1, 2, and 3, respectively. Lower scores indicated higher satisfaction with the major in question (Table 1).

According to the description and analysis of student satisfaction, the average satisfaction score for all students was 1.91. Students majoring in liberal arts were more satisfied with their majors than the overall average whereas students majoring in science, art, and sports were less satisfied.

To test whether major satisfaction scores differed between majors, we performed a one-way ANOVA of the three groups (liberal arts, science, and art and sports). The results showed a significant difference between the three majors (Table 2).

The post-hoc analysis indicated that there were significant differences in major satisfaction between liberal arts versus science and between liberal arts versus art and sports (Table 3).

### 4.2. Employment Preferences

The sequence of suitable employers for self-development after graduation, as selected by the undergraduate participants, was established, as shown in Table 4.

The top-choice employer for university students after graduation is a school. As per the overall results, the government, research institutions, and state-owned enterprises were more favored employers by students. This indicates that students are more inclined to seek professional, stable, and low-risk jobs when choosing employment, and this is more significant among female and liberal arts students. Female students ranked private companies, foreign-funded companies, and other challenging jobs in the same order. When choosing enterprises, male students were more willing to choose private enterprises than other enterprises, indicating that male students favored higher income and self-development opportunities but with a higher risk than female students.

### 4.3. Career Expectation

This study employed the career expectation scale developed by Wu [33]. The 22 career expectations were divided into three factors: F1 (prestige), F2 (self-development), and F3 (stability and welfare) (Table 5). F1 included eight projects relating to status, such as high employer visibility, ease of gaining distinction, starting a family, and opportunities for promotion. F2 included eight variables (further abbreviated using V and number) relating to the development of personal talents, such as being able to utilize one’s talents and undertaking challenging work. F3 included six variables relating to occupational stability and welfare. However, factor analysis was not implemented. Cronbach’s α coefficient of deleted items was 0.812–0.855, and Cronbach’s α coefficient of the overall scale was 0.847. The correlation coefficients of each dimension were 0.69–0.72.

The top five variables considered important by students in the ranking of career expectations were the ability to exert their talent (V7), career stability (V3), equality of opportunity and fair competition (V11), opportunity for education (V4), and high income (V1) (Table 6). Among the prestige factors, the item with the highest social status ranked 12th. This shows that students pay attention to whether they can utilize their talents and value self-development, followed by stability and welfare. However, students thought that factors relating to reputation and status, such as ease of gaining distinction (V19), high unit level (V20), overseas opportunities (V5), large unit size (V14), and high unit popularity (V13), were not very important.

### 4.4. Personal Evaluation and Social Support

Based on the personal evaluation and descriptive statistics, the average score of all students in the personal evaluation was 121.34 (Table 7). The score of male students was 115.69, which was lower than the average score of all students, and the average score of female students was 124.16, which was higher than the average score of all students. In terms of social support, the average score for all students was 39.29. The average score for males was 39.83, which was higher than the average score for all students. The average score for female social support was 39.03, which was lower than that of all students. This shows that females’ personal evaluations were higher than those of males, and males’ social support was higher than that of females.

The coefficients of the correlation analysis of personal evaluation, social support, major satisfaction, and career expectations were calculated. According to the results (Table 8), personal evaluations were significantly correlated with social support, major satisfaction, and career expectations. There was a significant negative correlation between personal evaluation and social support; students with higher personal revaluation values received less social support, while those with less social support tended to have a relatively high personal evaluation value. There was a significant positive correlation between personal evaluation, major satisfaction, and career expectations; that is, students with higher personal evaluations had higher satisfaction with their majors and held more optimistic career expectations. There was a significant negative correlation between social support, major satisfaction, and career expectations; that is, students with more social support were more dissatisfied with their majors and held less-than-ideal career expectations.

### 4.5. Correlation Analysis of Career Expectation Factors with Personal Evaluation and Social Support

Three factors (career expectations, personal evaluation, and social support) were analyzed. As can be seen from the results in Table 9, there was a significant correlation between F1 (prestige) and social support, with a correlation coefficient of 0.165; that is, the higher the prestige was, the higher the social support was. The correlation coefficient between F2 (self-development) and personal evaluation was 0.339, indicating a significant positive correlation. F3 (stability and welfare) was significantly correlated with social support, with a correlation coefficient of 0.174.

There are 54 items on the Personal Evaluation Questionnaire, graded on a four-point scale with an overall score range of 54-216. The evaluation scores were divided into three groups: a low-score group (54 ≤ X1 ≤ 108), middle-score group (109 ≤ X2 ≤ 162), and high-score group (163 ≤ X3 ≤ 216). According to the statistical results, the number of students in the middle-score group was the largest, accounting for 76.9% of all students. The number of students in the high-score group accounted for 25.1%, and the number of students in the low-score group accounted for approximately 2.00%.

The self-development factors in personal evaluation and career expectations were analyzed using ANOVA and multiple comparisons. As shown in Table 10, there was no difference in personal evaluation between the high-scoring and middle-scoring groups, while there was a significant difference between the low-scoring and middle-scoring groups. This indicates that differences in personal evaluations have a significant effect on self-development in career expectations.

The Social Support Scale consists of ten items with a total score ranging from 12 to 60 points. Social support scores were divided into three groups: a low group (12 ≤ Y1 ≤ 28), middle group (29 ≤ Y2 ≤ 44), and high group (45 ≤ Y3 ≤ 60). After multiple comparisons of the prestige factors and social support across the three groups (see Table 11), there were significant differences between the high-, middle-, and low-scoring groups, while there was no difference between the middle- and low-scoring groups, indicating that the level of social support had a significant impact on students’ career expectations concerning prestige.

Multiple comparisons were made between the levels of social support and the levels of stability and welfare in the participants’ career expectations, and the results are shown in Table 12. There were significant differences in the level of stability and welfare between the middle- and low-scoring social support groups, while there was no difference between the high- and low-scoring groups or the high- and high-scoring groups. This indicates that the amount of social support plays a significant role in the development of students’ career expectations concerning stability and welfare.

### 4.6. Regression Analysis of Personal Evaluation and Social Support on Career Expectation

This study found that college students’ personal evaluations and social support may affect their career expectations. To explain the influence of different factors on students’ career expectations based on correlation analysis, regression equations were introduced with students’ personal evaluation and social support as independent variables and the three factors of career expectations as dependent variables, and multiple regression analysis was conducted. The results are presented in Table 13.

The analysis indicated that personal evaluation and social support had predictive effects on college students’ career expectations. Therefore, this study hypothesized that there may be a causal relationship between personal evaluation, social support, and college students’ career expectations and that their internal composition differences can be investigated by establishing a path model. The independent variables were personal evaluation and social support. Taking the factors of prestige, self-development, and stability and welfare in students’ career expectations as dependent variables, the standardized partial regression coefficient β for the variables was obtained using path analysis and multiple linear regression. A path with a smaller coefficient was deleted using step-based regression, and social support was excluded from the model. For the stability and welfare factor, personal evaluation was excluded from the model, and for the self-development factor, neither personal evaluation nor social support were entered into the regression equation.

## 5. Discussion

### 5.1. Career Expectations for College Students of Different Majors and Genders

According to a sample survey of undergraduates at the Northeast Normal University and Anshan Normal University, 59.75% of students chose to work after graduation. It can be seen that the increasingly fierce employment situation prompts more students to find a job. Among them, 66% of the male students and 53.5% of the female students chose to work after graduation, while 26.8% of the male and 44.6% of the female students chose to continue studying. After graduation, the number of males who chose employment was higher than that of females, and the number of females who chose to continue studying was greater than that of males.

The students chose employers most suitable for their own development after graduation; the first choices of employers are schools, government agencies, scientific research institutions, state-owned enterprises, and other stable, low-risk, and good welfare employers. This ranking is more obvious among female and liberal arts students. This reflects fierce competition in the talent market and students’ lack of confidence in their abilities. Women ranked private enterprises, foreign-funded enterprises, and other challenging employers simultaneously. When choosing enterprises, male students were more willing to choose private enterprises, indicating that male students were more willing to favor higher-income and self-development opportunities but a higher risk than female students.

Students of liberal arts majors were more willing to choose employers with stable, high social status and good welfare. In contrast, science majors tended to favor research institutions. Art and sports majors showed greater flexibility and challenges in their choices of institutions. Private enterprises were the most popular, whereas scientific research institutions, state-owned enterprises, and foreign enterprises were not selected. Overall, when graduates chose employment, they focused on self-development through stable working conditions and good welfare benefits. Conversely, students preferred employers offering greater challenges and risks, such as private and foreign enterprises.

These results are mostly consistent with the findings of O’Connor et al., who suggest that millennials like jobs with security and fulfillment apart from just salaries [36]. The findings of Robinson et al. also indicate that when students consider career success, they also take into account the factors of benefits and meaningful work [25]. Our participants were expected to work as school teachers teaching their major subject areas. This was especially evident in the northeast of China, where the economy is not as well developed as compared to major cities such as Beijing. Holding a teaching position in a public school means they have a sustainable income and benefits despite not receiving a large salary. This might conflict with the expectations of parents and grandparents who, due to the one-child policy in China, have high hopes. The reason for these decisions may be due to the two universities not featuring in the top university rankings, and thus, the students are not as competitive as those from top universities both in the employment environment and in further study opportunities.

### 5.2. Analysis of Influencing Factors of College Students’ Career Expectations

According to the results of the Career Expectation Scale, the top five variables considered important by students were the ability to utilize their talents, career stability, equality of opportunity and fair competition, ability to provide educational opportunities, and high income. These are self-development factors (ability to use their own talents, fair competition in opportunity, and provision of educational opportunities) and stable welfare factors (career stability and high income). Among the prestige factors, the item with the highest social status ranked 12th. At present, the students generally focus on whether they can utilize their talents and develop self-factors, followed by stability and welfare factors. However, students think that factors such as the ease of earning distinction and starting a family, high level employers, opportunities to go abroad, large-scale employers, high visibility, and employer prestige are less important. This shows that students pay attention to self-development and materials and do not pursue “false fame” when choosing a career. The students prefer to focus on projects that can utilize their talent, career stability, fair competition, and educational opportunities. Males valued access to information and earning a high income. Females valued good welfare in line with their interests and a good medical pension. It can be seen that males pay more attention to income and meeting individual self-realization needs in their career expectations. In contrast, females pay more attention to variables that reflect the quality of personal life and the employer welfare, followed by high income.

Furthermore, 23.2% of the students were satisfied with their major and 62.9% were somewhat satisfied; only 13.9% of the students were not satisfied with their major. There were significant differences in the degree of satisfaction among students with different majors. Liberal arts majors had the highest degree of satisfaction, while art and sports students had the lowest degree of satisfaction. Most liberal arts and science majors rated their job prospects as “good” and “fair”, whereas only 40% of art and sports majors rated their job prospects as “poor”. One possible explanation for this phenomenon is that liberal arts students enrolled in these universities exhibit a relatively low rate of success in gaining admission to master’s programs. However, they demonstrate a notable track record of achieving teacher positions within the education system, which may contribute to their higher levels of satisfaction. There was a positive correlation between career expectations and major satisfaction, and the higher a student’s degree of satisfaction with their major was, the better their career expectations were. In the ranking of career expectations, students from all majors ranked “utilizing their talent” as the most important item. The ranking difference between art and science majors was not significant, focusing on career stability, medical pension funds, fair competition, information, and other variables. Liberal arts majors valued educational opportunities and welfare more than other students. Science majors rated factors such as high income and personal interest as more important. In terms of prestige, liberal arts majors ranked 14th and science majors ranked 3rd. Art and sports majors focused on fair and unfettered competition. This result is consistent with previous predictions regarding career expectations for different majors.

Notably, our results found that liberal arts majors showed the greatest satisfaction. This might be due to the situation, whereby our participants are mainly influenced by the local culture in northeast China. The older adults in these areas might consider the best jobs to be the most stable type. Furthermore, compared with other types of universities, the students from normal universities are expected to become teachers. As most students have a high chance of gaining teaching positions and have a very low success rate of becoming master’s students, most choose to find a job rather than having a master’s entrance exam. Additionally, liberal arts majors found it difficult to find a job other than teaching, which may have contributed to them having the highest degree of satisfaction.

### 5.3. Relationship between Personal Evaluation, Social Support, and Career Expectations

We found that under the influence of the traditional Chinese “son preference” concept, male students received more family and social support than female students, but the difference was not significant, indicating that gender equality is slowly progressing. The level of social support has a significant impact on the prestige of students’ career expectations. According to the results, students’ career selection is influenced by family and society, and students with more social support consider more prestigious career factors to meet their respective needs. The development of a stable welfare factor for students’ career expectations significantly influenced social support. Students with less social support consider more stability and welfare factors when choosing a career rather than satisfying material needs to realize their own value.

Further, personal evaluation had a predictive effect on prestige. Social support had a predictive effect on stability. Personal evaluations and social support can only partially explain college students’ career expectations. Other factors that may affect the career expectations of college students should be studied.

### 5.4. Implications and limitations

This study highlights the complex interplay of internal and external factors shaping students’ career outlooks. The influence of gender, major, self-concept, and social support highlight the need for nuanced, personalized career development interventions. By addressing gaps between student expectations and labor market realities, counselors can help manage unrealistic expectations and build career resilience. Supporting positive self-concepts and peer connections emerge as important factors for nurturing students’ career agency and adaptability. However, this finding might not reflect the whole situation of college students as it was only based on students from two normal universities. These students are more typically expected to become school teachers. Specifically, the level of Bachelor education at normal universities for most majors is aimed at training potential teachers for primary or middle schools. Therefore, such students might be more likely to choose jobs that are more stable. This is also influenced by significant differences in university rankings nationwide and the educational, local cultural, and employment environment the participants had experienced.

## 6. Conclusions

We found that most students believed their career expectations were satisfactory. Students majoring in science performed better than those majoring in liberal arts, art, and sports. A positive correlation was observed between career expectations and satisfaction. The higher the degree of satisfaction was, the better the career expectations were. Furthermore, the preferred employer for graduates was a school. According to the ranking of career expectation variables, students were willing to choose stable, low-risk, and good welfare units. Students majoring in art and sports demonstrated greater flexibility and challenges in unit selection. Meanwhile, when selecting a career, college students think that exerting their talent is the most important factor; the self-development is equally as important as stable welfare, and prestige is not important. In the career selection process, students lose their professional consciousness and focus on learning opportunities. Moreover, the level of personal evaluation had a significant effect on self-development in career expectations. There were significant differences in personal evaluations between males and females. Finally, the level of social support had a significant effect on the prestige, status, and welfare stability in career expectations.

## Figures and Tables

**Table 1:** Satisfaction with majors.

Major	Mean	SD
Liberal Arts	2.020	0.857
Science	1.800	0.628
Art and Sports	1.670	0.488
Overall	1.910	0.646

**Table 2:** Results of satisfaction scores: differences between majors.

	F	Sig
Group	3.780	0.025 *

* *p* < 0.05.

**Table 3:** Post-hoc analysis of major satisfaction scores.

Major	Major	Sig
Liberal Arts	Science	0.030 *
	Art and Sports	0.033 *
Science	Liberal Arts	0.030 *
	Art and Sports	0.455
Art and Sports	Liberal Arts	0.033 *
	Science	0.455

* *p* < 0.05.

**Table 4:** Employment sequence selected by participants.

Preferable Employment Unit Sequence						
Group	1	2	3	4	5	6
Overall	School	Government	Research institution	State-owned enterprise	Private enterprise	Foreign company
Males	School	Research institution	Government	Private enterprise	State-owned enterprise	Foreign company
Females	School	Government	Research institution	State-owned enterprise	Private enterprise	Foreign company
Liberal Arts	School	Government	Research institution	State-owned enterprise	Private enterprise	Foreign company
Science	School	Research institution	Government	State-owned enterprise	Private enterprise	Foreign company
Art and Sports	School	Government	Private enterprise			

Note: The number indicates the preferable employment unit sequence the participants chose, where 1 means the most preferable employment unit while 6 means the least preferable one.

**Table 5:** Career expectation factor analysis.

F1 (Prestige)			F2 (Self-Development)			F3 (Stability and Welfare)		
Variable	Content	Load	Variable	Content	Load	Variable	Content	Load
V20	High unit level	0.789	V11	Equality of opportunity and fair competition	0.662	V2	Benefits	0.774
V21	Unit in big cities	0.742	V18	Challenge	0.662	V16	Convenient transportation	0.747
V14	Large unit size	0.725	V22	Resource	0.615	V3	Career stability	0.701
V13	High unit popularity	0.718	V17	Freedom	0.604	V1	High income	0.666
V19	Esay Fame	0.716	V10	Interests	0.602	V8	Insurance	0.664
V5	Overseas opportunities	0.645	V7	Talents	0.505	V9	Good working environment	0.587
V6	Higher social status	0.623	V15	Apply what you learn	0.471			
V12	Promotion opportunities	0.584	V4	Opportunity of education	0.465			

**Table 6:** Career expectation variables’ importance ranking.

Ranking	Overall	Male	Female	Liberal Arts	Science	Art and Sports
1	V7	V7	V7	V7	V7	V7
2	V3	V22	V3	V3	V3	V11
3	V11	V1	V11	V4	V8	V17
4	V4	V3	V2	V2	V11	V22
5	V1	V11	V10	V1	V22	V4
6	V10	V4	V4	V10	V1	V3
7	V2	V10	V8	V11	V10	V10
8	V22	V2	V22	V22	V2	V1
9	V8	V8	V22	V8	V15	V2
10	V15	V15	V9	V15	V14	V8
11	V9	V17	V15	V9	V9	V15
12	V6	V21	V6	V6	V6	V12
13	V17	V18	V17	V12	V17	V9
14	V12	V9	V12	V21	V12	V21
15	V18	V16	V13	V17	V16	V18
16	V21	V12	V18	V18	V5	V6
17	V16	V6	V5	V13	V13	V14
18	V13	V14	V14	V16	V14	V16
19	V14	V20	V16	V14	V18	V13
20	V5	V5	V21	V5	V21	V20
21	V20	V13	V19	V20	V19	V5
22	V19	V19	V20	V19	V20	V19

**Table 7:** Personal evaluation and social support scores.

	Personal Evaluation		Social Support	
	Mean	SD	Mean	SD
Overall	121.34	19.156	39.29	6.046
Male	115.69	23.998	39.83	6.498
Female	124.16	15.595	39.03	5.83

**Table 8:** Correlation results of personal evaluation, social support, major satisfaction, and career expectation.

	Personal Evaluation	Social Support	Major Satisfaction	Career Expectation
Personal Evaluation	1	−0.440 **	0.318 **	0.327 **
Social Support	−0.440 **	1	−0.249 **	−0.198 **
Major Satisfaction	0.318 **	−0.249 **	1	0.357 **
Career Expectation	0.327 **	−0.198 **	0.357 **	1

** *p* < 0.01.

**Table 9:** Correlation results of career expectation factors with personal evaluation and social support.

	Personal Evaluation		Social Support	
	*R*	*p*	*R*	*p*
F1 (prestige)	0.139	0.093	0.165 *	0.046
F2 (self-development)	0.339 **	0.000	0.135	0.104
F3 (stability and welfare)	0.067	0.419	0.174 *	0.035

* *p* < 0.05, ** *p* < 0.01.

**Table 10:** Results of personal evaluation difference in terms of self-development.

	High Score	Middle Score	Low Score
High Score	-	0.469	0.564
Middle Score	0.469	-	0.000 **
Low Score	0.564	0.000 **	-

** *p* < 0.01. Note: Groups are divided according to the score of the personal evaluation. The results indicate the differences in self-development between groups.

**Table 11:** Results of social support differences in terms of prestige.

	High Score	Middle Score	Low Score
High Score	-	0.023 *	0.003 *
Middle Score	0.023 *	-	0.517
Low Score	0.003 **	0.517	-

* *p* < 0.05, ** *p* < 0.01. Note: Groups are divided according to the score of the social support. The results indicate the difference of the prestige factor between groups.

**Table 12:** Results of social support differences in terms of stability and welfare.

	High Score	Middle Score	Low Score
High Score	-	0.534	0.564
Middle Score	0.534	-	0.000 **
Low Score	0.252	0.000 **	-

** *p* < 0.01. Note: Groups are divided according to the score of the social support. The results indicate the difference of the stability and welfare factor between groups.

**Table 13:** Multiple regression analysis results.

	Prestige Factor	Stability and Welfare Factor
Personal Evaluation	0.278 **	
Social Support		−0.173 *
*R*2	0.078	0.03
*F*	12.100 **	4.450 *

* *p* < 0.05, ** *p* < 0.01.

## Data Availability

The data presented in this study are available on request from the corresponding author.
